# Comprehensive Studies of the Processes of the Molecular Transfer of the Momentum, Thermal Energy and Mass in the Nutrient Media of Biotechnological Industries

**DOI:** 10.3390/bioengineering9010018

**Published:** 2022-01-06

**Authors:** Aleksandr G. Novoselov, Sergei A. Sorokin, Igor V. Baranov, Nikita V. Martyushev, Olga N. Rumiantceva, Aleksey A. Fedorov

**Affiliations:** 1 Faculty of Biotechnologies, ITMO University, Kronverkskiy Prospekt, 49, 197101 St. Petersburg, Russia; aafedorov@itmo.ru; 2 Faculty of Energy and Ecotechnology, ITMO University, Kronverkskiy Prospekt, 49, 197101 St. Petersburg, Russia; SorokinSA@itmo.ru (S.A.S.); rumiantseva@itmo.ru (O.N.R.); 3 School of Biotechnology and Cryogenic Systems, ITMO University, Kronverkskiy Prospekt, 49, 197101 St. Petersburg, Russia; ivbaranov@itmo.ru; 4 Materials Science Department, Tomsk Polytechnic University, 30, Lenina Ave., 634050 Tomsk, Russia; martjushev@tpu.ru

**Keywords:** momentum transfer, dynamic viscosity, density, thermal diffusivity, thermal conductivity, beet molasses

## Abstract

This article puts forward arguments in favor of the necessity of conducting complex measurements of molecular transport coefficients that quantitatively determine the coefficients of dynamic viscosity, thermal diffusivity and molecular diffusion. The rheological studies have been carried out on the viscometers of two types: those with a rolling ball (HÖPPLER^®^ KF 3.2.), and those with a rotary one (Rheotest RN 4.1.). The thermophysical studies have been performed using the analyzer Hot Disk TPS 2500S. The measurements have been taken in the temperature range of 283 to 363 K. The concentration of dry substances has varied from 16.2 to 77.7% dry wt. An empirical equation for calculating the density of aqueous solutions of beet molasses has been obtained. The diagrams of the dependence of the dynamic viscosity on the shear rate in the range of 1 s^−1^ to 500 s^−1^ at different temperatures have been provided. The diagrams of the dependence of the coefficients of thermal conductivity and thermal diffusivity on the temperature and the concentration of dry substances have been presented, and empirical equations for their calculation have been obtained. The findings can be used for engineering calculations of hydrodynamic and heat-exchange processes in biotechnological equipment.

## 1. Introduction

Cultivation of microorganisms in production quantities is of great importance for biotechnological food production [1]. In particular, it is impossible to obtain high-quality bakery products or alcohol-containing products (alcoholic beverages) without the Saccharomyces cerevisiae yeast demanding the inclusion of yeast cells directly in the formulation of the initial ingredients [2]. The production of alcohol, wine and beer is also impossible without the participation of this kind of yeast cells at the fermentation stage [3,4]. In both cases, in the industrial production of the mentioned target products, their obtainment requires a constant reproduction of the biomass. The above-mentioned biomass is reproduced in special apparatuses—fermenters, which implement the process of aerobic submerged cultivation of microorganisms in a liquid nutrient medium by the air-inflow method [1].

To date, many different designs of fermenters have been developed [5], the classification of which is presented in works [1,6]. The generally accepted classification is that by the method of supplying energy to the fermenter [6], and the most common design of the fermenter used for aerobic cultivation of yeast cells in industrial-scale volumes is a bubble-type fermenter [7].

The process of aerobic cultivation of yeast cells is accompanied by a large release of heat energy, generated as a result of complex exothermic reactions inside the cell [8]. During the life of the cells, this heat must be removed to the surrounding cells, the environment, i.e., directly to the nutrient medium. From it the thermal energy in its turn is removed through various heat exchange devices (heating jackets or external heat exchangers of various types). The temperature at which the process of optimal cultivation of baker’s yeast cells is carried out is in a rather narrow range, within 28–32 °C for baker’s yeast and 23–30 °C for brewer’s yeast [9,10].

In this way, during aerobic cultivation of the microorganisms to increase the biomass, it is necessary to ensure that each cell is supplied with sufficient nutrition and respiration for its maximum growth rate and to create optimal conditions for their life activity, primarily temperature. In this case, it is necessary to take into account the fact that the concentration of the biomass during cultivation will constantly increase [8] and, therefore, the mass flows of nutrients and dissolved oxygen from the environment should also increase.

During metabolism of the increasing number of the cells, the amount of dissolved carbon dioxide, released by the cells into the liquid medium, will inevitably increase, which, later, diffusing through it into air bubbles, is removed from the fermenter. Mass flows of the oxygen, consumed by cells, and carbon dioxide, released by the cells, are approximately equal in the considered hour of cultivation.

The mechanism for the transfer of heat energy and mass largely depends on the hydrodynamic conditions in the working volume. The main resistance to the mass transfer rates and thermal energy at the molecular level is found in the liquid phase, and their values depend on the degree of its turbulization intensity [6].

On the surface of the separating phases, be it a solid phase or a gas phase, there are almost always boundary layers where the absence of turbulence is observed. The thickness of these layers can be commensurate with the sizes of the molecules. Even near the surface of the bubbles of the gas phase there is a layer of the liquid phase, conditioned by the presence of the surface tension of the liquid, surrounding the volume of the gas, contained in the bubble. This phenomenon is confirmed by numerous empirical equations, in which, as a rule, parameters are introduced that take into account the thicknesses of the laminar boundary sublayer, thermal sublayer or diffusion sublayer. The presence of these layers suggests that the transfer of the momentum (impulse), thermal energy and mass of the target component is significantly lower than that in the main volume of the working medium. And this occurs by the mechanisms of the molecular transfer, that is, viscous friction, thermal diffusivity and molecular diffusion. The values of these three mechanisms are quantitatively expressed by coefficients of kinematic viscosity ν, a coefficient of thermal diffusivity a and a coefficient of molecular diffusion $D_{\mathrm{AB}}$, respectively. There are currently no comprehensive studies aimed at measuring these coefficients. There are undoubtedly data on these values, but these measurements were performed more than 40 years ago [11] and the objects of study were different. In addition, in the meantime, the technology, hardware and equipment for producing sugar from sugar beets and, consequently, beet molasses, have changed significantly. Moreover, over this course of time, research techniques, methods and their instrumentation, related to the digital processing of measuring signals, as well as the obtained measurement results, have advanced significantly.

The culture media of microbiological industries have a complex chemical composition [12,13]. The main goal that is pursued when compiling a nutrient medium is the uninterrupted and balanced supply of cells with chemical elements, required for their successful life activity and maximum growth. The nutrient medium of the Saccharomyces cerevisiae yeast is based on water, enriched with beet or cane molasses, supplemented with nutrient salts and various stimulants [14,15]. In this way, the nutrient medium represents a multicomponent aqueous solution with certain physical and thermophysical properties, depending primarily on the quantitative and qualitative composition.

Viscosity can strongly influence the nature of the fluid flow. It is known [16] that the nature of the fluid flow can be different. In the general case, according to the nature of the flow, liquids are divided into two types—Newtonian and non-Newtonian [17,18]. In the first case, the fluid flow obeys Newton’s law (1):

(1) $$\tau =-\nu \frac{\partial ({\bar{U}}_{x}\cdot \rho )}{\partial y}=-\mu \frac{\partial {\bar{U}}_{x}}{\partial y}=\mu \dot{\gamma }$$

where τ is viscous shear or shear stress, N/m^2^;

${\bar{U}}_{x}$ is a time-averaged local fluid velocity in the x direction, m/s;

*y* is the values of the coordinates of neighboring layers, taken in the direction perpendicular to the direction of movement of the x layers, m;

ρ is the density of liquid, kg/m^3^;

ν is kinematic viscosity of the liquid, m^2^/s;

The viscosity of Newtonian fluids does not depend on the shear rate and its duration. These fluids are usually low-viscosity, one-component (pure) fluids such as water. The second case includes all other fluids, the flow of which can be summarized by the following law:

(2) $$\tau =\tau_{0}+K\;{\dot{\gamma }}^{n}$$

where $\tau_{0}$ is the yield point, N/m^2^;

K is the consistency index;

*n* is the flow behavior index.

Equation (2) describes the flow of non-Newtonian fluids and, in our opinion, is universal for all the fluids since with $\tau_{0}$ = 0 and with *n =* 1 the consistency index becomes equal to μ, and this equation is transformed into Equation (1).

On the other hand, equality $\tau_{0}$ = 0 raises doubts for the following reasons. The fulfillment of this equality assumes the absence of a liquid mass and, as a consequence, the absence of intermolecular forces of attraction and repulsion. In real liquids, these physical phenomena are inevitably present, but they have very small values and, at present, cannot be experimentally determined since there are no corresponding measuring devices. Thus far, no one has directly measured the mass of one molecule and the molecular forces of interaction, nor created a device that allows creating controlled forces and rates of shear. Calculations, based on the introduction of macroscopic values into them, in particular, molar volumes and masses, allowed us to estimate these values. Therefore, a priori, Equation (2) is universal from the viewpoint of ideas about the structure of the substances, in particular, liquids, from the standpoint of molecular kinetic theory. Hence, it follows that $\tau_{0}$ ≠ 0 and, at least $\tau_{0}$ > $\tau_{M}$, where $\tau_{M}$ is the resultant stress created by the forces of attraction and repulsion.

## 2. Materials and Methods

The study used the molasses, produced by the joint-stock company “Kombinat Pishchevyh Produktov” (St. Petersburg, Russia). The concentration of the dry substances (DS) in the original sample was determined using a refractometer and amounted to 77.7% dry wt. From the initial sample, five solutions of various concentrations in the range of 15 to 60% dry wt. were prepared by dilution with distilled water. The masses of the molasses and water for the preparation of solutions of a predetermined concentration were set in accordance with the following formulas:

$$m_{w}=\frac{V\cdot \rho_{m}\cdot \left(w_{1}{-w}_{2}\right)}{\rho_{m}\cdot {\;w}_{1}{/\rho }_{w}\;{-\;\rho }_{m}\cdot {\;w}_{2}{/\rho }_{w}{+w}_{2}}$$

$$m_{m}=\left({V\;-\;m}_{w}{/\rho }_{w}\right)\rho_{m}$$

where $m_{w}$ is the mass of distilled water, g; $m_{m}$ is the mass of the molasses of the initial concentration, g; *V* is the required volume of the resulting solution, mL; $\rho_{m}$ is the molasses density of the initial concentration, g/cm^3^; $\rho_{w}$ is the water density, g/cm^3^ (taken equal to 1 g/cm^3^); $w_{1},w_{2}$ are initial and final mass fractions of DS.

The content of the solids in the obtained solutions was controlled using a refractometer. The density of the initial sample and solutions was determined using hydrometers.

The HÖPPLER^®^ KF 3.2 rolling-ball viscometer was used to measure the viscosity of solutions of all the concentrations. The measurements were carried out in a temperature range of 283–353 K. Before each experiment, the sample, poured into the sinking tube of the viscometer and was thermostated for 20 min to reach the specified temperature. Each experiment at a given temperature included 5 experiments, the results of which were used to determine the average time of the ball rolling in the sample.

In addition, for the samples with a DS concentration between 60.2 and 77.7% dry wt., the experiments were carried out to measure the viscosity on a Rheotest RN 4.1 rotary viscometer. The measurements were carried out in a temperature range of 283–343 K and in a shear rate range of 1–500 s^−1^. For each experiment, a 30 mL sample was taken into the measuring cell, after which the sample was thermostated for 20 min to reach the specified temperature. The duration of one experiment was 10 min–1 min per shear rate value.

**Figure 1:**
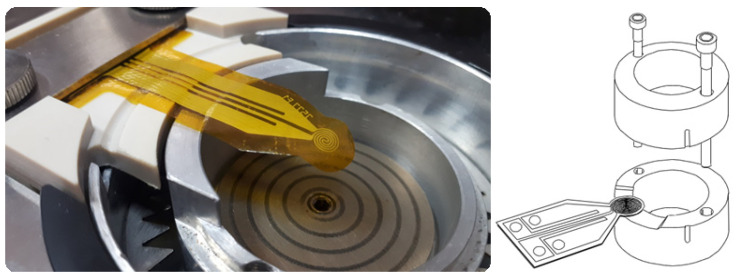
A general view of the kapton sensor and its location in the measuring cell.

The Hot Disk TPS 2500S analyzer was used to measure the thermophysical characteristics of beet molasses solutions for various concentrations. The measurements of this device are based on the transient plane source method [19]. It consists in the use of a special sensor, located in the center of the sample volume. The sensor consists of an electrically conductive grid in the form of the double helix, etched from a thin metal foil. This helix is placed between two thin films of insulating material (kapton). Before starting the experiment, the sample and the sensor were located inside the measuring cell, which is hermetically fixed with two screws (Figure 1). The experiment consisted in recording the temperature change inside the sample with a known heat flux. In this way, the thermal conductivity and thermal diffusivity were determined from a single registration of the transient process.

The measurements were carried out in the temperature range of 293–363 K with a step of 10 K. To reach the specified temperature, before each experiment, the solution placed in the sample holder was stabilized for some time until the temperature difference was within the deviation range of ±0.5 K. Each experiment at a specified temperature included five measurements, the results of which were used to determine the average value of the parameters under study.

## 3. Results

The results of measuring the density of the aqueous solutions of the beet molasses are presented in Table 1.

**Figure 2:**
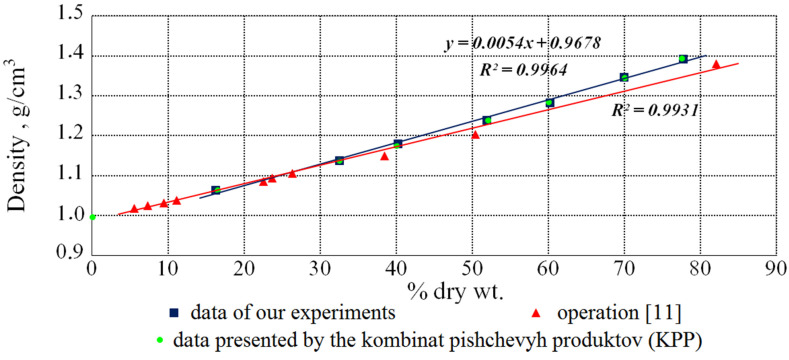
The dependence of the density of aqueous solutions of beet molasses on the content of dry substances at 293 K.

Based on the data obtained as a result of the measurements, dependence diagrams of the density on the content of the dry substances in the solutions under study were constructed for each investigated temperature. As expected, these dependencies were linear. An example is the diagram shown in Figure 2.

Figure 2 show that the data, obtained in our experiments, are in good agreement with the KPP data and, in the future, we were guided by them. The mathematical processing of the results, presented in this diagram, allowed us to obtain an empirical equation:

(3) $$\rho =5{.4\;\times \;10}^{-3}\cdot DS+9{.68\;\times \;10}^{-1}$$

where *DS* is taken in percent.

**Figure 3:**
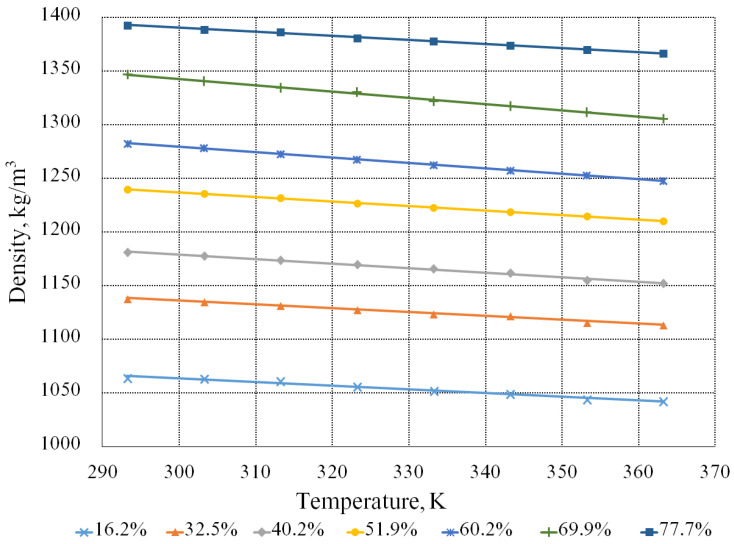
Dependence of the density of the aqueous solutions of the molasses on the temperature and content of the dry substances.

The dependence of the density of the aqueous solutions of the beet molasses on temperature is shown in Figure 3.

Figure 3 demonstrates that with an increase in the concentration of the dry substances in the solution, the density becomes greater, and with an increase in the temperature, the density, on the contrary, decreases. Let us note that a change in the temperature influences the density insignificantly relative to a change in the concentration of dry substances. The influence of the dry substances content on the density is about 12 times greater than the influence of the temperature. This is indicated by the coefficients with the corresponding parameters in empirical Formula (4) for calculating the density, obtained as a result of mathematical processing:

(4) ρ=1100 − 0.43·T+5.257·n

where *T* is the temperature, K;

*n* is the dry substances content, %.

The maximum relative deviation of this formula and experimental data is about 1%.

### 3.1. The Investigations of the Flow of Aqueous Solutions of the Beet Molasses

Based on the data, obtained as a result of rheological studies, the following dependence diagrams were built: flow curves, temperature-viscosity curves and influence curves of the dynamic viscosity coefficient on the shear rate for solutions with the dry substances concentration of 60.2% and 77.7%, as well as the dependence of viscosity on the dry substances content at different temperatures.

**Figure 4:**
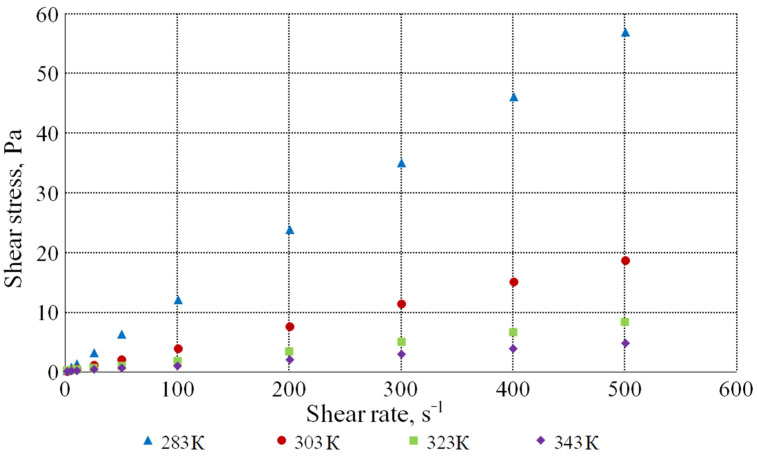
Dependence of the shear stress on the shear rate at different temperatures for the solution with the dry substances concentration of 60.2%.

Figure 4 shows the dependence of the shear stress on the shear rate at different temperatures for the solution with the dry substances concentration of 60.2%. The diagram shows that this dependence is linear and passes through the origin of coordinates, which characterizes this molasses solution as a Newtonian liquid.

**Figure 5:**
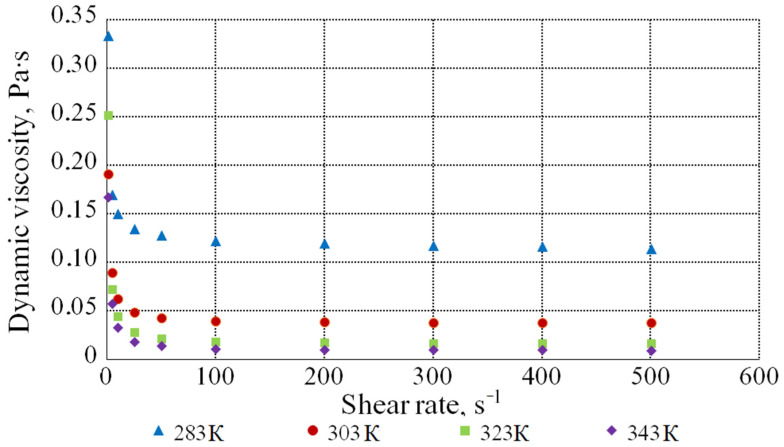
Dependence of the viscosity of the molasses solution with the DS content of 60.2% on the shear rate at different temperatures.

Figure 5 illustrates the dependence of the dynamic viscosity of the molasses solution with the DS content of 60.2% on the shear rate at different temperatures.

The value of the dynamic viscosity coefficient decreases with an increase in the shear rate from 1 s^−1^ to 50 s^−1^ and, with its further increase, it remains practically constant. Hence, at low shear rates, the solution behaves as a pseudoplastic liquid, and at rates above 50 s^−1^—as a Newtonian liquid.

**Figure 6:**
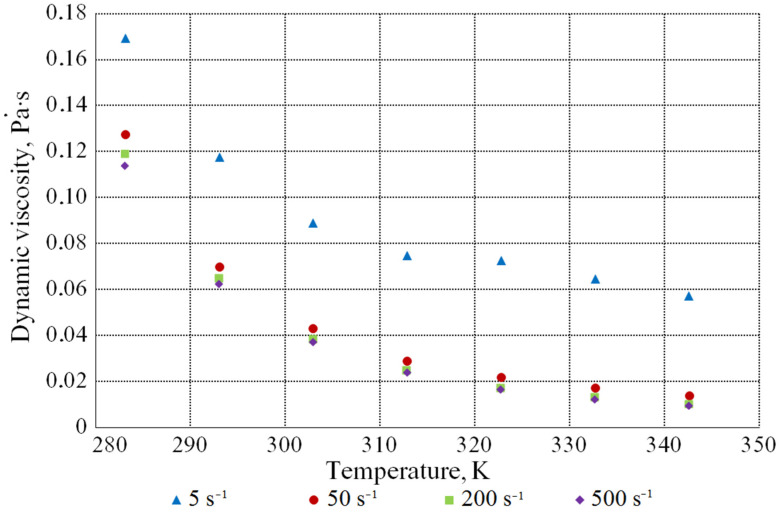
Dependence of the value of the dynamic viscosity coefficient on the temperature at different shear rates (DS content is 60.2 mass%).

Figure 6 shows the dependence of the value of the dynamic viscosity coefficient on the temperature at various shear rates. As the temperature rises, the molasses viscosity decreases.

**Figure 7:**
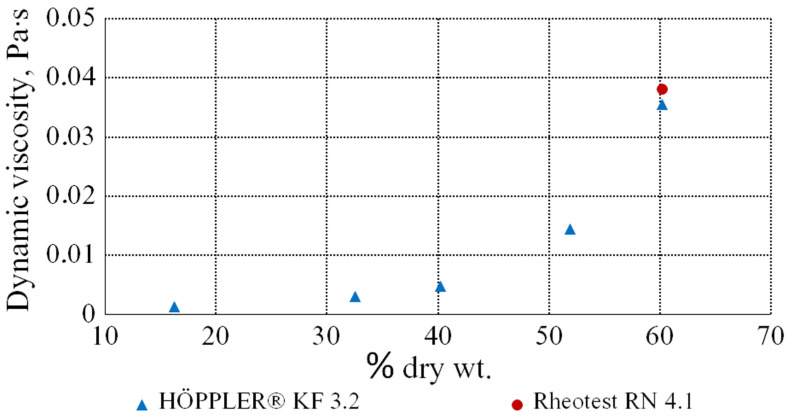
Dependence of the dynamic viscosity coefficient of the molasses solutions on the dry substances concentration (DS).

Figure 7 shows the dependence of the dynamic viscosity coefficient of the molasses solutions on the content of the dry substances at a temperature of 303 K. The values of the dynamic viscosity coefficients of the aqueous solutions of the beet molasses increase when the amount of DS increases.

This diagram is based on the data, obtained as a result of measuring the viscosity of the molasses solutions, using a rolling ball viscometer. In addition, a red dot is plotted on the diagram, representing the shear rate of 300 s^−1^, obtained as a result of measurements on the rotational viscometer. In this way, comparing the values, obtained on these devices, we can conclude that they give similar and, as a consequence, reliable results of the solution viscosity with a concentration of 60.2% dry wt. at a temperature of 303 K.

### 3.2. The Investigations of Thermophysical Properties of the Aqueous Solutions of the Beet Molasses

As a result of the measurements, data were obtained that allowed plotting the dependence diagrams of coefficients of the thermal conductivity and thermal diffusivity of the beet molasses solutions on the temperature with different contents of dry substances.

**Figure 8:**
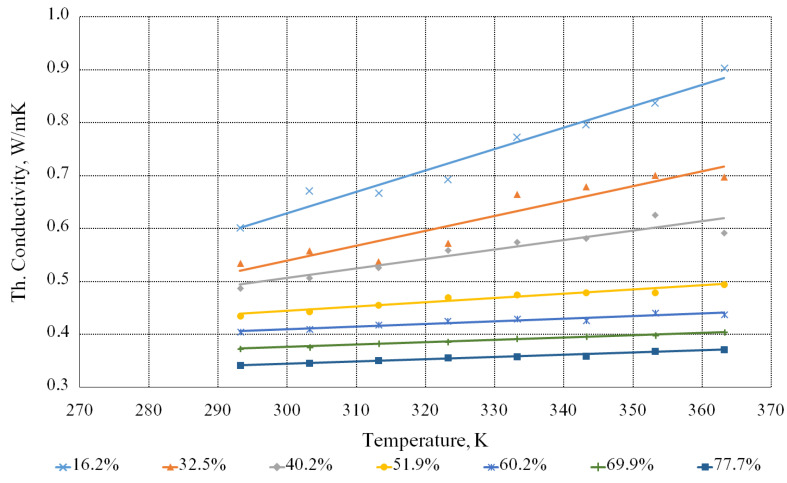
Dependence of the thermal conductivity coefficient of the aqueous solutions of the beet molasses on the temperature with different contents of the dry substances.

Figure 8 demonstrates the dependence of the thermal conductivity coefficient of the beet molasses solution on the temperature with different contents of the dry substances. The diagram shows that when the dry substances content increases in the solution, the thermal conductivity coefficient decreases and when the temperature increases, it, on the contrary, increases. That is, the lower the dry substances content in the solution and the higher the temperature, the higher the thermal conductivity coefficient. Let us note that when the temperature rises, the intrinsic energy of molecules of the molasses solutions increases, due to which the amplitude of oscillations increases and, as a result, thermal conductivity increases. However, this decreases their density and the force interaction of liquid molecules with each other. This leads to a decrease in thermal conductivity.

Since the dependence is linear, the equation for calculating the thermal conductivity coefficient of the beet molasses solution is presented by the following formula:

(5) $$\lambda \cdot 10^{4}=3062+15.83\cdot T-63.40\cdot n$$

This formula is valid for *T* values, ranging from 293 to 363 K, and *n*—from 16.2% to 77.7%. The maximum relative deviation is about 11%.

**Figure 9:**
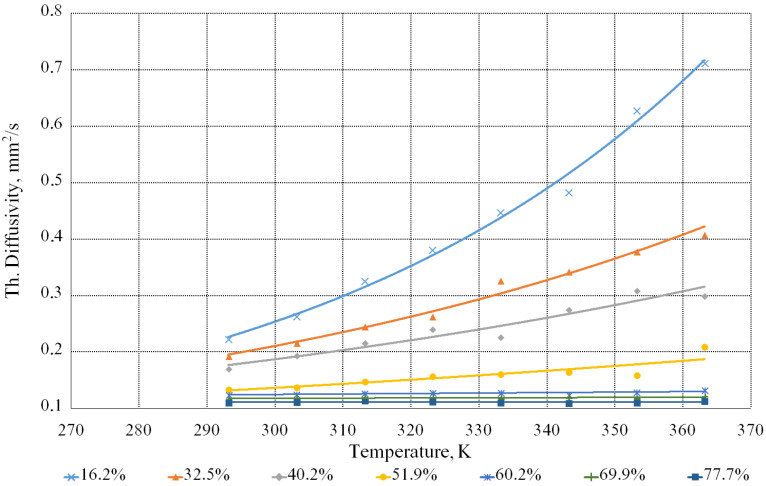
Dependence of the thermal diffusivity coefficient of the beet molasses solutions on the temperature with different contents of the dry substances.

Figure 9 illustrates the dependence of the thermal diffusivity coefficient of the beet molasses solutions on the concentration of the dry substances and the temperature.

The experimental data processing allowed us to obtain the calculated formula for the thermal diffusivity coefficient:

(6) $$a=0.08\;\cdot \;1.006^{T}\;\cdot \;0.978^{n}.$$

This formula is valid for *T* values, ranging from 293 to 363 K and *n*—from 16.2 to 77.7%.

The values of the parameters, obtained from the above-mentioned formulas, allow using the ratio for calculating the values of the specific heat capacity, connecting the thermophysical characteristics of any materials, including products.

$$C_{p}=\frac{\lambda }{\rho \cdot a}$$

This ratio allows calculating the specific heat of the beet molasses solutions. Specific heat is a physical quantity that is used in generation of heat balance equations. They are the basis for further calculations of heat and mass transfer equipment, including in industries, using the molasses solutions as raw feedstock [20]. Therefore, the studies, aimed at obtaining experimental and calculated data about the nature of the deviation in this value from various parameters are relevant and are of paramount importance for solving applied technological problems of such industries.

The influence of the dry substances’ concentration in the aqueous solutions of the beet molasses and the temperature on the coefficients of molecular diffusion of oxygen and carbon dioxide will be presented in a new paper. At this stage, the problem of obtaining a reliable equation for calculating this coefficient in clean water is considered. The results of this work can be found in [21,22].

## 4. Conclusions

On the basis of the experimental studies of the beet molasses solutions, the values of density, thermal conductivity and thermal diffusivity have been obtained in a wide range of temperatures and dry substances content, and the mathematical relations have been determined for their calculation. The nature of the flow of molasses aqueous solutions with different dry matter content is analyzed. Numerical values of dynamic viscosity coefficients are obtained depending on temperature variation and concentration of dry substances. It has been established that dependences *ρ* *= f(T, n)*, *λ = f(T, n)* are linear, and the dependence *a = f(T, n)* is indicative, proving that, when the concentration of dry substances decreases and the temperature increases, it is illustrated more clearly. The obtained equations can be recommended to determine the numerical values of density, thermal conductivity and thermal diffusivity for calculating the specific heat, solving applied technological problems of industries and using molasses solutions as raw feedstock.

## Figures and Tables

**Table 1:** Density values of aqueous solutions of the beet molasses with different contents of dry substances in the temperature range of 283–353 K.

Solution Density, g/cm^3^								
% Dry wt., K	283	293	303	313	323	333	343	353
**16.2**	1.068	1.064	1.063	1.061	1.056	1.052	1.049	1.044
**32.5**	1.141	1.138	1.135	1.132	1.128	1.124	1.122	1.116
**40.2**	1.185	1.181	1.178	1.174	1.17	1.166	1.162	1.155
**51.9**	1.247	1.24	1.236	1.232	1.227	1.223	1.219	1.215
**60.2**	1.285	1.283	1.279	1.273	1.268	1.263	1.258	1.253
**69.9**	1.351	1.347	1.341	1.335	1.331	1.322	1.318	1.312
**77.7**	1.405	1.393	1.389	1.387	1.381	1.378	1.374	1.37

## Data Availability

Not applicable.
